# Simultaneous Amperometric Aptasensor Based on Diazonium Grafted Screen-Printed Carbon Electrode for Detection of CFP10 and MPT64 Biomarkers for Early Tuberculosis Diagnosis

**DOI:** 10.3390/bios12110996

**Published:** 2022-11-09

**Authors:** Muhammad Hafiznur Yunus, Nor Azah Yusof, Jaafar Abdullah, Yusran Sulaiman, Nurul Hanun Ahmad Raston, Siti Suraiya Md Noor

**Affiliations:** 1 Institute for Research in Molecular Medicine (INFORMM), Universiti Sains Malaysia, Gelugor 11800, Pulau Pinang, Malaysia; 2 Institute of Nanoscience and Nanotechnology (ION2), Universiti Putra Malaysia, Serdang 43400, Selangor, Malaysia; 3 Department of Chemistry, Faculty of Science, Universiti Putra Malaysia, Serdang 43400, Selangor, Malaysia; 4 School of Biosciences and Biotechnology, Faculty of Science and Technology, Universiti Kebangsaan Malaysia, Bangi 43600, Selangor, Malaysia; 5 School of Medical Sciences, Universiti Sains Malaysia, Kubang Kerian 16150, Kelantan, Malaysia

**Keywords:** aptasensor, aptamer, dual detection, CFP10, MPT64, diazonium salt, amperometry, tuberculosis

## Abstract

Early diagnosis is highly crucial for life-saving and transmission management of tuberculosis (TB). Despite the low sensitivity and time-consuming issues, TB antigen detection still relies on conventional smear microscopy and culture techniques. To address this limitation, we report the development of the first amperometric dual aptasensor for the simultaneous detection of *Mycobacterium tuberculosis* secreted antigens CFP10 and MPT64 for better diagnosis and control of TB. The developed sensor was based on the aptamers–antibodies sandwich assay and detected by chronoamperometry through the electrocatalytic reaction between peroxidase-conjugated antibodies, H_2_O_2_, and hydroquinone. The CFP10 and MPT64 aptamers were immobilized via carbodiimide covalent chemistry over the disposable dual screen-printed carbon electrodes modified with a 4-carboxyphenyl diazonium salt. Under optimized conditions, the aptasensor achieved a detection limit of 1.68 ng mL^−1^ and 1.82 ng mL^−1^ for CFP10 and MPT64 antigens, respectively. The developed assay requires a small sample amount (5 µL) and can be easily performed within 2.5 h. Finally, the dual aptasensor was successfully applied to clinical sputum samples with the obtained diagnostic sensitivity (*n* = 24) and specificity (*n* = 13) of 100%, respectively, suggesting the readiness of the developed assay to be used for TB clinical application.

## 1. Introduction

Tuberculosis (TB) is regarded as one of the most prevalent infectious diseases globally, with approximately 9.9 million new cases reported in 2020 [1]. It is an airborne disease, thus easily contagious even by coughing, spitting, sneezing, or just talking [2]. TB commonly affects the lungs (pulmonary TB) but may also progress to other parts of the body, such as the spine, brain, kidney, and many other organs. The latter form of TB is addressed as extrapulmonary TB. TB commonly presents with prolonged cough, fever, loss of appetite, and night sweats; in the worst scenario, it can even result in death if untreated [3]. An estimated 1.3 million deaths were reported in 2020, increasing 8.3% compared to the previous year [1]. Thus, a proper diagnosis, treatment, and prevention mechanism should be critically identified to appropriately contain the disease and reduce the mortality rate caused by the infection.

Until now, smear microscopy has been the most frequently employed technique for active TB diagnosis due to its relatively low cost and rapidness in obtaining the result. That technique, however, suffers from a lack of sensitivity and specificity [4,5]. On the other hand, the bacteriological identification method is considered the gold standard for TB confirmation; although highly sensitive, the turnaround time is unacceptably slow; it may take up to 8 weeks, which causes a significant delay in the treatment plan. Therefore, these techniques still seem insufficient for global TB eradication efforts. Other molecular-based determination techniques are too expensive and unsuitable for routine use, especially in limited resources regions [6].

Electrochemical biosensors have attracted a lot of attention as a promising biodiagnostic application in recent years, including infectious diseases such as TB [7,8,9,10,11]. The advancement in electrochemical technology has resulted in highly sensitive detection assay with properties such as portability, non-laborious procedure, easy to miniature, low-cost, and low-volume sample requirements. These characteristics have made it among the outstanding potential solutions to improve the current TB diagnosis, especially for the development of point-of-care testing. The aptamer integration during the sensor’s fabrication has further extended the feasibility of the electrochemical biosensor in producing sensitive and specific performance in TB diagnosis. Many aptamers with different targets have been constructed and applied on TB biosensors, such as Ag85A [12], IFN-γ [13,14], CFP10 [8,15], MPT64 [16,17,18], and even the whole H37R cell [10,19] in efforts to improvise the TB diagnosis.

As expected, the developed aptamer-based diagnostic assays have shown excellent analytical performance in some down to femto-level target analyte detection. Despite demonstrating superior analytical performance, the review of previous studies still revealed significant gaps in TB diagnosis, particularly in their clinical diagnostic performance. For example, an impedance biosensor developed by Sypabekova et al. has successfully demonstrated a magnificently low detection limit of 4.1 fM of the target analyte; however, it only produced 76.5% diagnostic sensitivity when tested with a clinical sample [20]. Zhu et al. also found low diagnostic sensitivity of their developed biosensor of 64.4% despite having a reasonable detection limit [21]. Moreover, many other studies only reported the use of spiked samples as proof of concept for diagnostic application [16,17,22], which only indicated the hypothetical diagnostic application of their developed biosensor and may not represent the actual diagnostic performance without the clinical studies.

In order to address these limitations, a combination of more than a single biomarker in a detection assay has been explored as a promising approach for obtaining highly sensitive and specific diagnostic tools. The combination can be either from a direct mixture as a cocktail or engineered at the molecular level to form a fusion or chimeric molecule. Previous reviews have agreed that using combination molecules as the detection elements produce superior diagnostic performance compared to their correspondence single antigen [23,24,25,26]. The study by Souza et al. (2012) concluded that the combined ESAT6/MPB70/MPB83 antigens used in their enzyme immunoassay demonstrated superior diagnostic performance to ESAT-6 or MPB83 individually [25]. Similar results are reported in another study investigating IFN-γ response after stimulation to several TB antigens, i.e., single or multiple antigens. Their findings concluded that the response of samples exposed to combinations of antigens resulted in better diagnostic sensitivity compared to a single ESAT6 antigen (66%) in the following order ESAT6/MPT64 (89%) > ESAT6/CFP10 (73%) > 38 kDa/CFP10 (70%) [24].

Thus, taking advantage of the abovementioned approaches, the present study describes a novel electrochemical TB diagnosis that utilizes aptamers that simultaneously recognize two different biomarkers, i.e., CFP10 and MPT64, during detection. The sandwich configuration was applied where the target antigen was captured by the immobilized aptamer and the secondary peroxidase-labeled antibody probe. The performance of the developed technique was assessed using the patient’s sputum samples that were clinically validated by smear microscopy and bacteriological culture assays. The method described herein offers a high sensitivity and specificity technique with a simple fabrication and can easily be converted into a portable device, and has the potential to be implemented as a point-of-care diagnosis to facilitate the current TB management program.

## 2. Materials and Methods

### 2.1. Reagents

CFP10 and MPT64 aptamers were obtained from Integrated DNA Technologies (IDT, Coralville, IA, USA). *M. tuberculosis* CFP10 and MPT64 recombinant antigens, MPT64 and CFP10 peroxidase-labeled secondary antibodies were purchased from Cusabio (Wuhan, China). Human serum, bovine serum albumin (BSA), hydrochloric acid, 4-aminobenzoic acid, 4-morpholineethanesulfonic acid (MES), 1-ethyl-3-(3-dimethylaminopropyl)-carbodiimide (EDC), potassium hexacyanoferrate (III) (K_3_Fe(CN)_6)_, potassium hexacyanoferrate (II) trihydrate (K_4_Fe(CN)_6_·3H_2_O), hydroquinone (HQ), N-hydroxysuccinimide (NHS), phosphate-buffered saline (PBS), sodium chloride, sodium nitrite, and tris-hydrochloride were from Sigma-Aldrich (St. Louis, MO, USA). Calcium chloride, potassium chloride, magnesium chloride, and hydrogen peroxide (H_2_O_2_) were from R&M Chemicals (Essex, UK). All of the reagents were of analytical grade and used without further purification. Ultrapure water (18.2 MΩ·cm^−1^) was used throughout the study.

### 2.2. Instrumentation

The electrochemical measurements were performed using a μStat 8000 portable potentiostat equipped with DropView 8400 Software (DropSens, Asturias, Spain). Disposable dual screen-printed carbon electrode (DRP-X1110, Dropsens, Oviedo, Spain), consisting of two ellipses-shaped carbon ink working electrodes (6.3 mm^2^ each), a carbon ink counter electrode and a silver pseudo-reference electrode were used as the transducer.

A ThetaLite100 instrument (Biolin Scientific, Finland) was used to analyze the contact angle of the modified electrode. An Axis Ultra spectrometer (Kratos Analytical, Manchester, UK) was used to employ X-ray photoelectron spectroscopy (XPS) analysis with both survey scan and detailed scan by passing 160 eV and 20 eV energy, respectively. The raw data were then interpreted using CasaXPS software (version 2.3.25 PR1.0) with charge correction performed by referring to the hydrocarbon component of the C1s peak at 285.0 eV.

### 2.3. Preparation of Dual Aptasensor Based on the Carboxyphenyl-Modified Carbon Electrode

**Figure 1:**
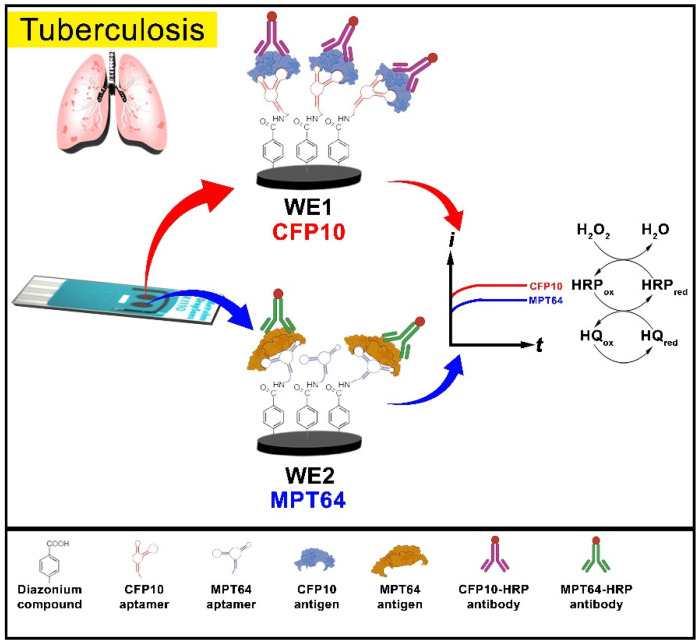
Schematic diagram of the preparation of the dual CFP10 and MPT64 aptasensor.

The in situ grafting of the 4-aminobenzoic diazonium salt was performed exactly as reported in our previous study [27]. The grafted diazonium film was then incubated with 100 mM EDC and 25 mM NHS prepared in MES buffer for 1 h at room temperature to activate the carboxylic group. The electrode was then washed with deionized water and allowed to air-dry. An amount of 2.5 µL of CFP10 and MPT64 capture aptamer diluted in binding buffer was dropped onto the diazonium-modified working electrodes and incubated at room temperature for 1 h. The working electrode 1 (WE1) of the dual-screen printed electrode was designated for CFP10 detection while working electrode 2 (WE2) was prepared for MPT64 antigen detection, as illustrated in Figure 1. The aptamer-immobilized surface was then rinsed with binding buffer, followed by 1 h incubation with ethanolamine solution. Finally, the electrode was blocked with 1% of BSA to prevent non-specific binding that would affect the sensor specificity. After a washing step with binding buffer, the electrode was ready for the detection analysis or can be kept at 4 °C for storage.

### 2.4. Characterization of Dual-Carboxyphenyl Diazonium Fabricated Electrode

The formation of the grafted diazonium film was confirmed by XPS analysis. The survey and high-resolution, detailed scan were performed on bare carbon electrode, diazonium-grafted carbon electrode, and after the immobilization of the captured aptamer to analyze the presence of different elements on the electrode surface. The contact angle measurements and analysis were also performed using a sessile drop technique based on Young’s equation.

The stepwise modifications were characterized with the EIS technique in 5 mM [Fe(CN)_6_]^3−/4−^ solution containing 0.1 M KCl at a frequency range of 100 kHz to 0.01 Hz and 5 mV amplitude. The Nova software version 2.1 was used during the analysis and the fitting of the impedance data.

### 2.5. Simultaneous Detection of CFP10 and MPT64 Antigens

The detection assay was performed by incubating the BSA-backfilled electrode with a range of CFP10 and MPT64 antigens for 1 h at room temperature. For the selectivity study, the electrodes were also incubated with the non-target proteins, i.e., MPT64 antigen (for WE1), CFP10 antigen (for WE2), human serum, and BSA solution. The electrodes were then rinsed, and peroxidase-labeled antibodies were dropped on the respective working electrode and further incubated for 1 h at room temperature. The electrodes were then rinsed, air-dried, and subjected to amperometric measurement in supporting electrolytes containing PBS (pH7.4), 2.0 mM H_2_O_2_, and 0.1 mM HQ at a potential of −0.2 V (vs. Ag pseudo-reference).

The experimental conditions for aptamers and peroxidase-labeled antibodies were optimized to achieve optimal performance when tested with the respective target antigens. In all optimizations, the electrodes were treated with 10 ng mL^−1^ control target antigen (S) and blank sample (buffer solution without target antigen) (B), and the conditions that generated the highest signal-to-blank (S/B) ratio were regarded as the optimum criteria.

### 2.6. Clinical Samples

Ethical approval for the patient’s sputum samples was obtained from the Medical Research and Ethics Committee, Ministry of Health Malaysia [NMRR-17-3001-39473 (IIR)]. The clinical samples employed in this study were obtained from the Institute of Respiratory Medicine, Ministry of Health, Malaysia. Sample collection was conducted according to the approved protocols, and informed consent was obtained from all participants before specimen collection. The samples were categorized into two groups, i.e., TB positive (TB (+), *n* = 24) and TB negative (TB (–), *n* = 13), according to their standard reference (smear microscopy and culture) results. TB (+) samples were confirmed positive with both smear microscopy and bacteriological culture, while the TB (–) samples were considered the non-disease control with negative results from both smear microscopy and culture assays. The collected samples were processed on the same day for the detection study.

### 2.7. Statistical Analysis

GraphPad Prism software version 9.2.0.332 (GraphPad Software, San Diego, CA, USA) was utilized for the statistical analyses of the experimental data. One-way ANOVA followed by Tukey’s multiple comparison test was performed on the data from the selectivity studies to determine the significant difference between the group means. The diagnostic sensitivity and specificity of the aptasensors were assessed using receiver operating characteristic (ROC) curve analysis. The optimum cut-off current reading was obtained according to the maximum value of the Youden index analysis (sensitivity + specificity – 1). Mann–Whitney test was performed to compare the statistical difference between TB (+) and TB (–) groups for the clinical study. All of the experimental data were based on the measurements with at least three replicates and presented as the average of replicates and standard deviation from the average value. In all statistical studies, a *p*-value < 0.05 was considered statistically significant.

## 3. Results

As described in the methodology section, the dual aptasensor was based on a sandwich enzyme-linked assay utilizing both aptamer and antibody as biorecognition molecules. Adapted from our previous works on the individual detection of each tuberculosis antigen [8,27], the dual aptasensor was prepared by covalent attachment of the respective CFP10 and MPT64 aminated aptamers on the diazonium-modified carbon surfaces, which are rich with carboxyl terminal. The blocking step with ethanolamine and BSA introduced afterward helped to lower the background signal from the non-specific molecule binding. After placing the samples (or standard solutions) onto the respective electrode surface, the peroxidase-labeled secondary antibodies were added to the electrode, forming a sandwich configuration of the aptamer-target antigen-antibody complex. The twice-analyte recognition by the capture aptamer and later by the secondary antibody can significantly improve the specificity of the developed assay [28]. Figure 1 displays the fabrication process involved in the development of the diazonium-based dual CFP10 and MPT64 aptasensor.

The target antigens were determined by amperometric measurement of H_2_O_2_ responses mediated by HQ at a potential of –0.2 V (vs. Ag pseudo-reference). The HRP-H_2_O_2_-HQ is a renowned detection technique employed in many diagnostic applications for many reasons. HRP reagent can be easily acquired due to its high availability and also a low-cost reagent [29,30]. In addition, a wide range of substrates can be paired and oxidized by HRP for detection analysis, thus making HRP a reagent for choice in enzymatic detection reactions. The presence of the HQ in the detection system can improve the detection response since the reduction process of the enzymatic reaction is generally slow due to slow electron transfer. Thus, HQ can accelerate the electron transfer process between the surface and the HRP redox center [27,31,32].

### 3.1. Characterization of the Diazonium-Modified Dual Aptasensor

**Figure 2:**
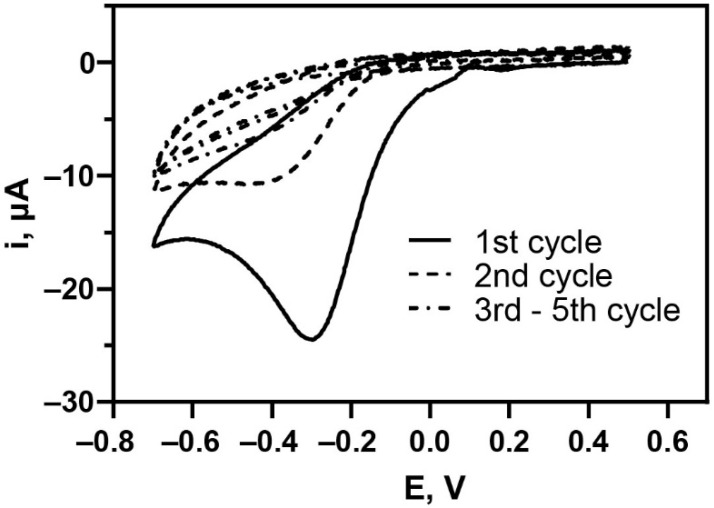
CV voltammograms of the in situ electrografting of 4-aminobenzoic acid diazonium compound on the dual screen-printed carbon electrode surface.

The covalent attachment of the carboxyphenyl diazonium on the carbon surface through the electroreduction technique resulted in the formation of a typical diazonium reduction peak on the CV voltammogram, as shown in Figure 2. The broad irreversible signal was observed at a potential of around −0.3 V in the first CV cycle, representing the aminobenzoic diazonium precursor reduction by a single electron transfer mechanism. A nitrogen molecule was eliminated from the precursor reagent during the reaction to form an aryl radical ready to be covalently grafted onto the surface afterward. The reductive current signal was significantly reduced upon the continuous CV sweeping as the growth of aryl species blocked the conductive carbon surface. This phenomenon suggests the effective binding of the diazonium species on the electrode surface, which prevents additional reduction of more diazonium cations on the electrode [33,34]. The intensity of the reductive current turns out to be negligible starting from the third CV cycle onwards, indicating the significant aryl diazonium coverage on the carbon surface during the first and second cycles. Thus, two CV cycles have been employed for electrografting the carboxyphenyl diazonium compound during the dual aptasensor development.

The diazonium-modified electrode was then proceeded to contact angle measurement to confirm the presence of the diazonium film on the carbon surface. Since the grafted diazonium film was rich in the carboxylic acid functional group, which showed a high hydrophilic characteristic, the comparison with the bare carbon electrode that is hydrophobic in nature was expected to result in a significant contact angle difference between both surfaces. The mean measurement of at least five replicates for the diazonium-modified carbon surface was 50.4 ± 1.6°; which decreased significantly compared to the bare carbon surface of 118.6 ± 0.6°. The wetting enhancement of the diazonium film can be explained by the presence of a high density of carboxyl functionality due to the formation of covalent binding of the carboxyphenyl diazonium on the carbon surface. The representative contact angle images are shown in Figure S1 (Supplementary Materials).

**Figure 3:**
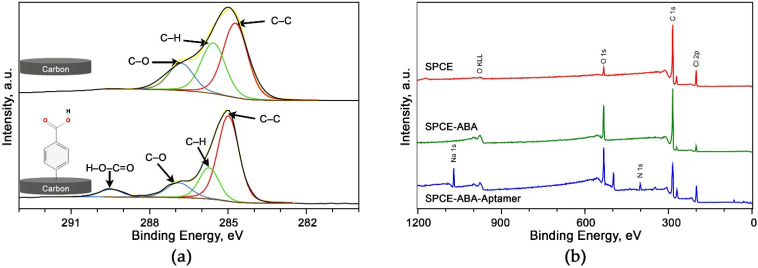
Characterization of the fabricated dual aptasensor (**a**) Carbon 1s spectra analyses comparison between the bare carbon electrode and diazonium grafted surface (**b**) XPS survey scan spectra for bare, diazonium-modified, and after aptamer immobilization.

The result was further supported by the XPS finding that demonstrates the evidence of the carboxylic functionality from the diazonium-modified surface. Figure 3a shows the high-resolution C1s spectra of the bare carbon electrode and electrode after fabrication with the diazonium salt. The deconvoluted C 1s spectra resulted in three common components for bare and diazonium-modified electrodes. On both surfaces, a strong asymmetric and narrow peak was present at approximately 284.9 eV, a characteristic of C–C graphitic carbon [35]. Both electrodes also exhibited a shoulder peak at the higher binding energy (286.7 eV) that corresponds to C–O groups. Another peak positioned at around 285.7 eV was also noticed on both surfaces, ascribed to the C–H or C–C aliphatic bond components [36]. A peak at around 289.5 eV attributed to the COOH appeared on the spectra of the diazonium-modified surface but not on the bare carbon surface. There is also a notable increase in O1s signal on the survey scan of the diazonium-modified electrode (Figure 3b) compared to the unmodified carbon electrode. This phenomenon was due to an increased number of oxygen atoms in the carboxyl functionality of the diazonium compound (in this case, the oxygen signal is proportional to carboxyl density) [37]. Thus, those findings confirmed the presence of the diazonium compound on the electrode surface.

### 3.2. Electrochemical Characterization of Diazonium-Modified Dual Aptasensor

**Figure 4:**
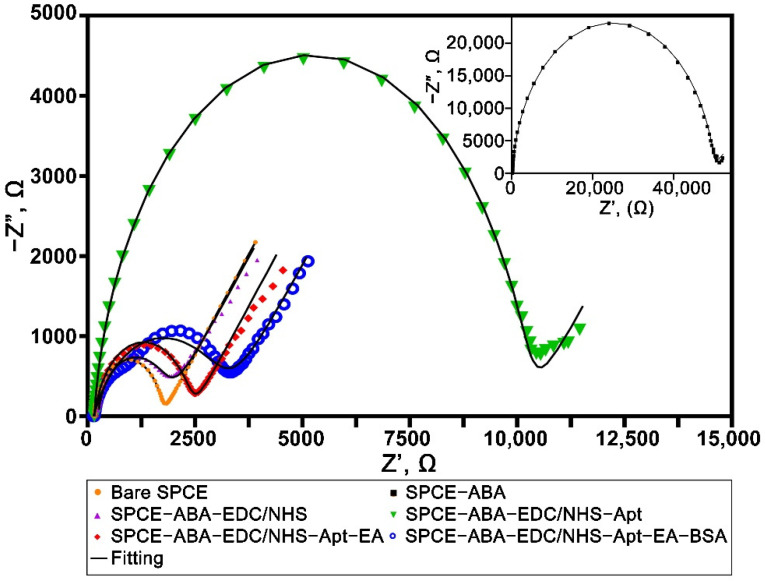
Nyquist plot of the stepwise fabrication of the aptasensor performed in 5 mM [Fe(CN)_6_]^3−/4−^ containing 0.1 M KCl at the frequency ranging from 100 kHz to 0.01 Hz. Inset shows the Nyquist plot of the diazonium-modified surface.

The step-by-step fabrications were monitored with EIS in a 5 mM [Fe(CN)_6_)]^3−/4−^ redox probe. Figure 4 illustrates the Nyquist plot for every step involved during the assembly of the WE1 of the aptasensor. The plots were then fitted to the equivalent circuit, as suggested in Figure S2 (Supplementary Materials). An expected small semicircle was observed on the bare electrode, implying the low impedance value (R_ct_) due to the free access of the conductive carbon surface to the redox probe. There was a massive R_ct_ increase in the electrode upon attachment of the diazonium film from 1568.1 Ω to 49,371 Ω caused by electrostatic repulsion from the negatively charged carboxyl group. The subsequent EDC/NHS activation step decreased the impedance value to 1562.2 Ω due to the neutralization of the negatively charged carboxyl group from the surface, allowing more interaction with the [Fe(CN)_6_)]^3−/4−^ redox probe. The R_ct_ value showed an increase after the aptamer’s immobilization on the electrode’s surface, most likely due to the repulsive barrier resulting between the negatively charged nucleotide structure of the aptamer and the (Fe(CN)_6_]^3−/4−^ redox probe. The successful attachment of the aptamer was also supported by the XPS analysis, which showed the appearance of the N1s peak on the survey scan spectra, as illustrated in Figure 3b. The nitrogen element originated from the amide bonding observed on the electrode after immobilizing the aminated aptamer on the diazonium film and was not present on bare and diazonium-modified surfaces. The R_ct_ value dropped to 2224.5 Ω upon the deactivation of the carboxyl-terminal, most probably due to the replacement of loosely-binding aptamer on the electrode surface. The final blocking stop with BSA increased the impedance value because of the formation of the dense surface coverage by the protein that acts as a kinetic barrier hindering the electron transfer between the redox probe and electrode surface. Similar behavior was observed in the impedance results of WE2 (not shown) since the same materials were used during the modification steps. The detailed values of the impedance parameters obtained over the EIS fitting are shown in Table S1 (Supplementary Materials).

### 3.3. Optimization of the Aptasensor Variables

**Figure 5:**
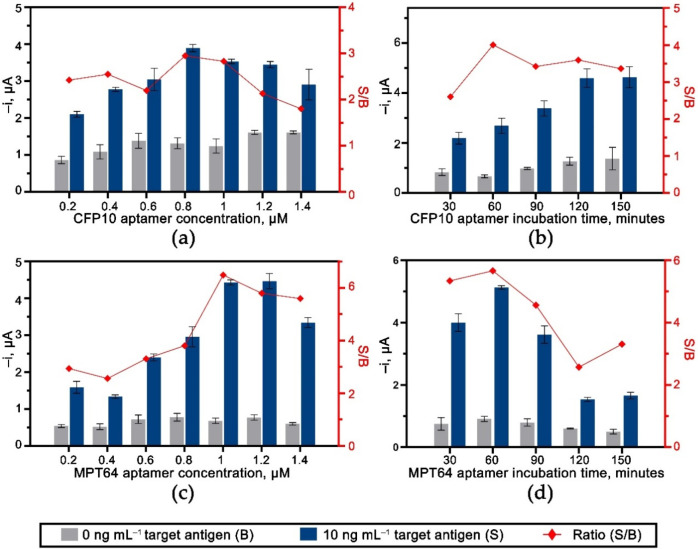
The effect of aptamers loading and their incubation time for both (**a**,**b**) WE1 (CFP10) and (**c**,**d**) WE2 (MPT64) (*n* ≥ 3).

The effects of the experimental variables for the dual CFP10 and MPT64 aptasensor were studied for each target antigen. The optimized parameters include the concentration and the incubation time for both capture aptamers and antibodies on the dual aptasensor. Figure 5 shows the results of the optimization performed on the aptamers immobilized on the diazonium-modified surface. As for CFP10 aptamer conditions, Figure 5a,b indicates that the most significant S/B current ratios were at 0.8 µM and 60 min for the concentration and incubation time, respectively. The amperometric current dropped when more than 0.8 µM CFP10 aptamer was loaded on the electrode surface, probably because of the growth of a crowd and densely packed surface that limited the binding efficiency between the aptamer and the CFP10 target antigen [17,27]. A slightly higher aptamer concentration (1 µM) was chosen as the best condition for the MPT64 sensor since it produced the highest S/B current ratio (Figure 5c). A similar incubation duration of 60 min was set as an optimum for MPT64 aptamer immobilization, as depicted in Figure 5d.

**Figure 6:**
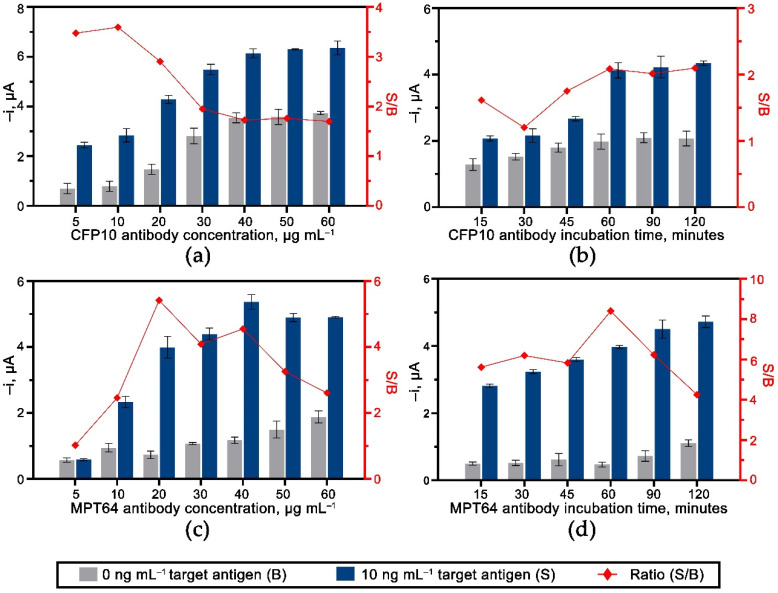
The optimization of concentration and incubation time for the secondary HRP-antibodies for (**a**,**b**) WE1 (CFP10) and (**c**,**d**) WE2 (MPT64) (*n* ≥ 3).

The effect of different antibody loading for both CFP10 and MPT64 working electrodes are presented in Figure 6a,c, respectively. An increasing trend of the amperometric currents was observed with the increased antibody concentration up to 40 µg mL^−1^ for both optimizations. The current signals were then decreased (MPT64) or showed no significant changes (CFP10) with a higher concentration (>40 µg mL^−1^) of antibody loaded during the assay. As expected, there was also an increase in the non-specific signals with increased antibody concentration employed during the detection due to the higher amount of non-specific antibodies attached to the electrode surface. Thus, 10 (CFP10 antibody) and 20 µg mL^−1^ (MPT64 antibody) were selected for further work since they produced the highest S/B ratio. CFP10 and MPT64 antibodies also showed adequate incubation at 60 min duration with an acceptable non-specific response, as illustrated in Figure 6b,d.

**Figure 7:**
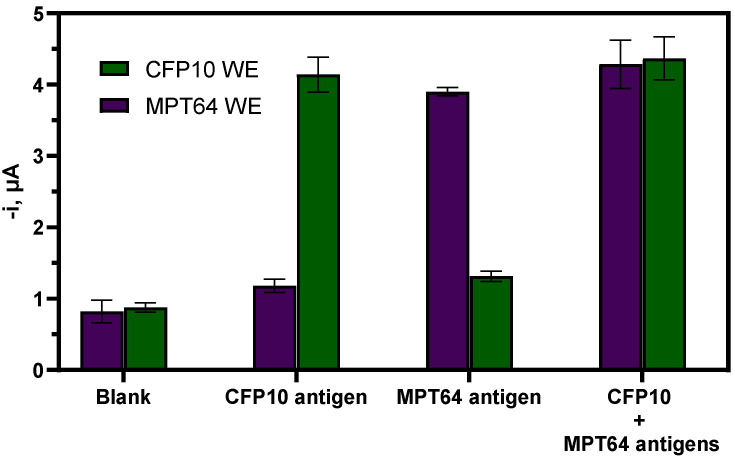
Amperometric responses of the simultaneous dual aptasensor measured with different mixtures of CFP10 and MPT64 recombinant antigens; Blank (0 ng mL^−1^ of both antigens); CFP10 antigen (10 ng mL^−1^ CFP10 antigen and 0 ng mL^−1^ MPT64 antigen); MPT64 antigen (0 ng mL^−1^ CFP10 antigen and 10 ng mL^−1^ MPT64 antigen) and CFP10+MPT64 antigens (10 ng mL^−1^ CFP10 antigen and 10 ng mL^−1^ MPT64 antigen) (*n* = 3).

Another important consideration in the preparation of dual or multiplex biosensors is ensuring no cross-talking between the neighboring modified working electrodes. Thus, the possible cross-reaction between the WE1 (CFP10) and WE2 (MPT64) in this work was assessed with different CFP10 and MPT64 antigens mixtures (Figure 7). The findings clearly demonstrated no significant cross-talking between the electrodes, thus supporting the viability of the developed dual aptasensor for tuberculosis detection.

### 3.4. Analytical Performance of the CFP10 and MPT64 Dual Aptasensor

**Figure 8:**
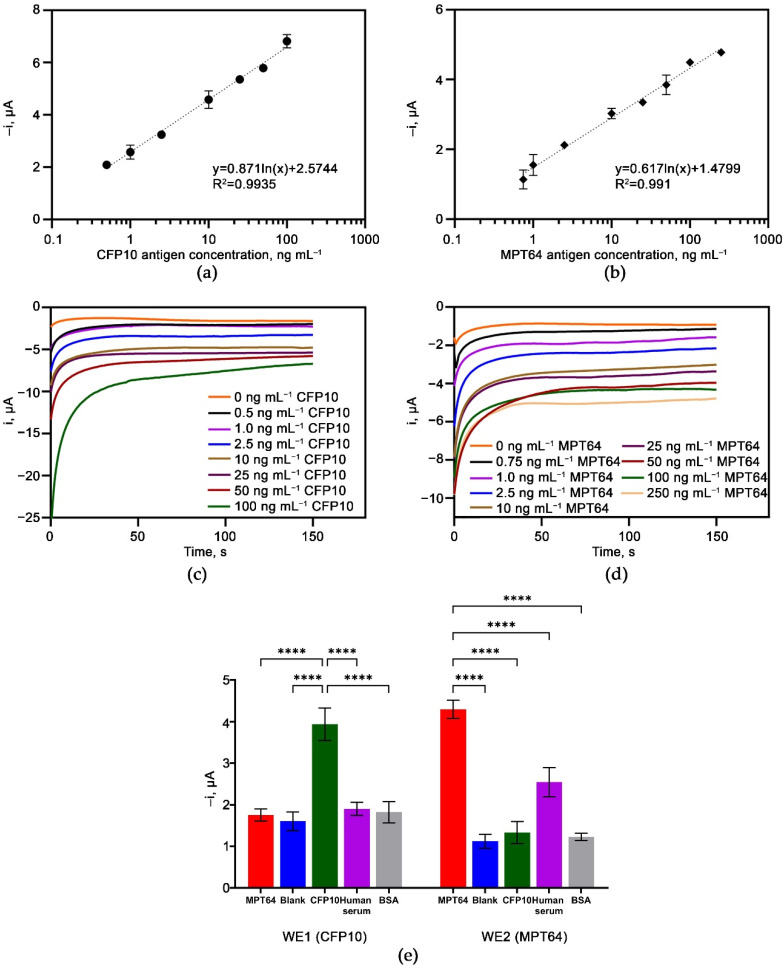
Amperometric responses of the dual aptasensor incubated with a series of (**a**) CFP10 and (**b**) MPT64 antigens. Chronoamperograms of different concentrations of (**c**) CFP10 antigen and (**d**) MPT64 antigen. (**e**) Selectivity study of the dual aptasensor. Statistical analysis was performed using the post hoc Tukey test. Data are based on the mean of at least three replicates ± standard deviation; **** denotes *p* < 0.0001.

The developed aptasensor was incubated with a series of CFP10 and MPT64 target antigen concentrations under optimized conditions to determine its analytical performance. The calibration plot of both antigens, as shown in Figure 8a,b, exhibited a linear relationship with the correspondence target antigen concentration in the range of 0.5–100 ng mL^−1^ for CFP10 and 0.75–250 ng mL^−1^ (MPT64) with R^2^ of 0.994 and 0.991, respectively. Figure 8c,d show that the reduction current gradually increases with increasing concentration of CFP10 and MPT64 antigen, respectively. The limits of detection (LOD) values were determined according to the 3 σ/*m* formula, where σ was the standard deviation of the amperometric responses of the blank solution and *m* was the slope of the corresponding calibration plot. The obtained LODs were 1.68 ng mL^−1^ and 1.82 ng mL^−1^, respectively. The calculated limits of quantification (LOQs), derived from the 10 σ/*m* formula, were estimated to be 5.66 ng mL^−1^ and 7.70 ng mL^−1^ for CFP10 and MPT64, respectively.

The LODs achieved in this study are comparable to those in previous studies on the detection of tuberculosis. The method is obviously better in terms of analytical performance when compared to the traditional enzyme-linked immunoassays (ELISA) method, as performed by Liu and colleagues, which only produced a detection limit of 10 ng L^−1^ for the detection of MPT64 antigen [38]. A previous work by Mohd Azmi on detecting CFP10 antigen using an antibody managed to obtain a detection limit of 15 ng mL^−1^ [39]. Later, they further improved their study by replacing the antibody with an aptamer as the capture molecule prepared on the same fabrication configuration and found a better detection limit of 1.5 ng mL^−1^ [40] that was on par with the LODs achieved in this work. Their finding also corroborates the idea of applying aptamer as a biorecognition element, as utilized in the current work, to improve the analytical performance of the developed biosensor. Furthermore, compared to their work, this study employed a simpler fabrication technique with less material and steps during fabrication, thus lowering the assay’s production cost and making it feasible for point-of-care testing, especially for those who stay in resource-limited settings. Another study by Chutichetpong and co-workers focused on the drug susceptibility test using MPT64 as a biomarker and produced a slightly lower LOD value of 0.43 ng mL^−1^. They employed a similar amperometric detection technique with different reagents of the catalytic reaction of 3,3′-5,5′-tetramethylbenzidine (TMB), H_2_O_2_, and HRP [41].

### 3.5. Selectivity and Reproducibility Study of the Aptasensor

The developed dual aptasensor was assessed with various non-target reagents to evaluate its selectivity performance, as shown in Figure 8c. The findings indicated that the developed dual aptasensor was selective only towards its corresponding target but not with the other non-target reagents. However, the aptasensor should be used with caution if serum samples are being employed since there was a noticeable current increase from the human serum on the WE2 sensor. Except for the latter, all other non-target reagents showed current signals below the obtained LODs values. As the catalytic reaction can only occur with the presence of the peroxidase tagged on the secondary antibody probe, the signal obtained from the non-target reagents must come from the non-specific binding of the HRP-tagged antibody on that reagent. Further improvement in future work is necessary to lower the background signal as it may shadow the target responses, especially when the target antigen is present in a trace amount. Nonetheless, the statistical analysis has proven insignificant of all signals for the non-target reagents with the *p*-value < 0.0001.

The reproducibility of the dual aptasensor was evaluated by measuring the amperometric responses for the five different electrodes incubated with 10 ng mL^−1^ CFP10 and 25 ng mL^−1^ MPT64 antigen prepared on the same day. The dual aptasensor showed good reproducibility with RSD values of 2.6% and 2.99% for CFP10 and MPT64, respectively.

### 3.6. Simultenoues Detection of CFP10 and MPT64 in Clinical Sputum Samples

**Figure 9:**
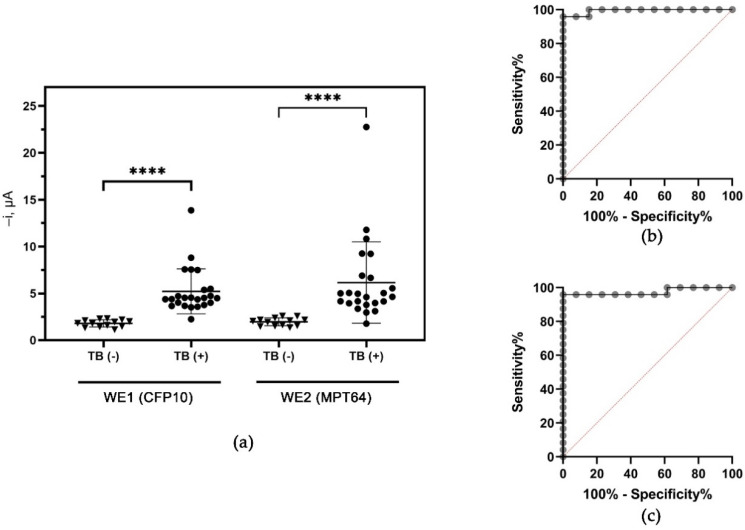
Clinical diagnosis of sputum samples from individuals infected with tuberculosis (TB(+)) and non-tuberculosis individuals (TB(–)). (**a**) Tabulation of amperometric current response of TB (+) and TB (–) samples tested with the dual aptasensor. Group comparisons were analyzed using Mann–Whitney test. **** represents *p* < 0.0001. ROC analysis of TB (+) and TB (–) samples on the (**b**) WE1 (CFP10) and (**c**) WE2 (MPT64).

The dual CFP10 and MPT64 aptasensor were used to evaluate the clinical sample obtained from individuals diagnosed with pulmonary tuberculosis labeled as TB (+) and individuals without the disease (TB (–)). The results presented in Figure 9 show the ability of the aptasensor to discriminate between the TB (+) and TB (–) samples on both working electrodes with *p* values < 0.0001, analyzed using a non-parametric Mann–Whitney U-test analysis (Figure 9a). The optimum cut-off values, which produce the highest diagnostic sensitivity with minimal specificity loss, were predicted using ROC analysis. The cut-off amperometric current values suggested by the ROC were –2.926 µA for WE1 resulting in a diagnostic specificity of 100% (95% CI: 77.19 to 100.0%) and 95.8% (95 CI: 79.76% to 99.79%) sensitivity. As for WE2, a similar 100% specificity (95% CI: 77.19 to 100.0%) was obtained with a slightly lower sensitivity of 91.7% (95% CI: 74.15 to 98.52%), based on the cut-off at –3.040 µA suggested by the ROC analysis. The ROC also corroborates the diagnostic potential of the dual aptasensor with AUC values of 0.9936 (95% CI: 0.9768 to 1.000) and 0.9744 (95% CI: 0.9228 to 1.000) for CFP10 and MPT64 working electrodes, respectively.

**Figure 10:**
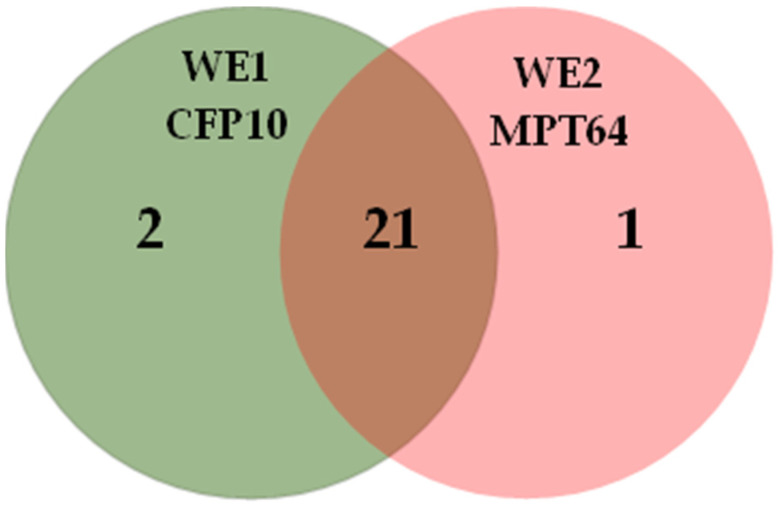
Venn diagram of the distribution of the positive results (*n* = 24) obtained with the simultaneous CFP10 and MPT64 dual aptasensor.

The summary of the number of positive results tested with TB (+) samples is summarized in a Venn diagram, depicted in Figure 10. CFP10 sensor (WE1) performed better than the MPT64 sensor (WE2), with 23 out of 24 TB (+) samples detected. On the other hand, the MPT64 sensor (WE2) correctly identified 22 TB (+) samples, with two samples interpreted as false negative results. Interestingly, three samples (CFP10 = 2; MPT64 = 1) were uniquely detected by the dual aptasensor, highlighting the significance of having two instead of a single biorecognition element in an assay. The combined results from both working electrodes provided a 100% diagnostic sensitivity and specificity, which performed better compared to some of the assays developed in previous studies, as presented in Table 1. These findings are in agreement with previous work that emphasizes the importance of having more than a single reagent as a biorecognition element [26,42,43]. Raja and colleagues observed a significantly improved sensitivity from 67% to 89% when the combined biomarkers (30 kDa, 16 kDa, and 38 kDa) were employed in their ELISA assay. The obtained sensitivity of the single antigens ranged from 57% to 67% [43]. Yunus et al. also reported using three different bioreceptors on their lateral flow immunoassay, which increased the parasitic infection’s diagnostic sensitivity [26].

A limitation of this work is that it only assessed a limited number of sputum samples; thus, further clinical evaluation studies recommended in multicenter with higher sample numbers are necessary to validate the diagnostic value of the developed dual aptasensor. Furthermore, the sample from different matrices, i.e., serum, biopsies, or cerebrospinal fluid, can also be used to test the applicability of the dual aptasensor on extrapulmonary tuberculosis patients.

## 4. Conclusions

This work presents a novel electrochemical aptasensor for the simultaneous detection of two potential biomarkers in tuberculosis, CFP10 and MPT64. The aptasensor offers a simple but strongly stable diazonium-based modification on the surface of a disposable screen-printed electrode. The combination of two distinct aptamers as recognition elements produced an excellent 100% diagnostic sensitivity (*n* = 24) and specificity (*n* = 13). The assay has a good potential to be further utilized as a point-of-care test that benefits the people living in the endemic area for a better patient diagnosis. The outstanding preliminary performance of the dual aptasensor merits further evaluation using a larger number of samples.

## Figures and Tables

**Table 1:** Summary of the selected electrochemical biosensors for TB detection.

Detection Technique	Target	Linear Range	LOD	Diagnostic Performance		Reference
				Sensitivity	Specificity	
DPV	CFP10-ESAT6	5–500 ng ml^−1^	1.5 ng mL^−1^	100% (*n* = 6)	100% (*n* = 4)	[40]
DPV	CFP10-ESAT6	10–500 ng mL^−1^	1.5 ng mL^−1^	100% (*n* = 6)	91.7% (*n* = 11)	[44]
EIS	MPT64	0.1 fM–1 nM	4.1 fM	Sputum: 76.47% Serum: 88.24% (*n* = 17)	Sputum: 100% Serum: 100% (*n* = 4)	[20]
DPV	HspX	13 pM–648 nM	13 pM	92.3% (*n* = 13)	91.2% (*n* = 57)	[45]
DPV	*M. tuberculosis* DNA	0.01–100 ng µL^−1^	0.05 ng μL^−1^	–	–	[46]
DPV	CFP10	20–100 ng mL^−1^	15 ng mL^−1^	–	–	[39]
CA	CFP10 and MPT64	CFP10: 0.5–100 ng mL^−1^ MPT64: 0.75–250 ng mL^−1^	CFP10: 1.68 ng mL^−1^ MPT64: 1.82 ng mL^−1^	CFP10 WE: 95.8% MPT64 WE: 91.7% Overall, CFP10+MPT64: 100% (*n* = 24)	CFP10 WE: 100% MPT64 WE: 100% Overall, CFP10+MPT64: 100% (*n* = 13)	This work

DPV: differential pulse voltammetry, EIS: electrochemical impedance spectroscopy, CA: chronoamperometry.

## Data Availability

Not applicable.

## Supplementary Materials

The following supporting information can be downloaded at: https://www.mdpi.com/article/10.3390/bios12110996/s1, Figure S1: Contact angle analysis of (a) bare carbon electrode and (b) diazonium grafted surface; Figure S2: Suggested equivalent circuit model of the Nyquist plot from EIS measurements. Rs: solution resistance; Rct: charge-transfer resistance; W: Warburg impedance; CPE: constant phase element; Table S1: Fitting parameters for Nyquist plot for stepwise modification of the developed sensor.
