# Supplementary material for: Simultaneous Amperometric Aptasensor Based on Diazonium Grafted Screen-Printed Carbon Electrode for Detection of CFP10 and MPT64 Biomarkers for Early Tuberculosis Diagnosis
Source: Biosensors (Basel). 2022 Nov 9;12(11):996. doi: 10.3390/bios12110996 (PMC9688523; doi:10.3390/bios12110996)
Supplement: Supplementary file 1 [file biosensors-12-00996-s001.zip › biosensors-2006910-supplementary.pdf]

biosensors

MDPI

Supplementary

Simultaneous Amperometric Aptasensor Based on Diazonium Grafted Screen-Printed Carbon Electrode for Detection of CFP10 and MPT64 Biomarkers for Early Tuberculosis Diagnosis

Muhammad Hafiznur Yunus, Nor Azah Yusof, Jaafar Abdullah, Yusran Sulaiman, Nurul Hanun Ahmad Raston and Siti Suraiya Md Noor

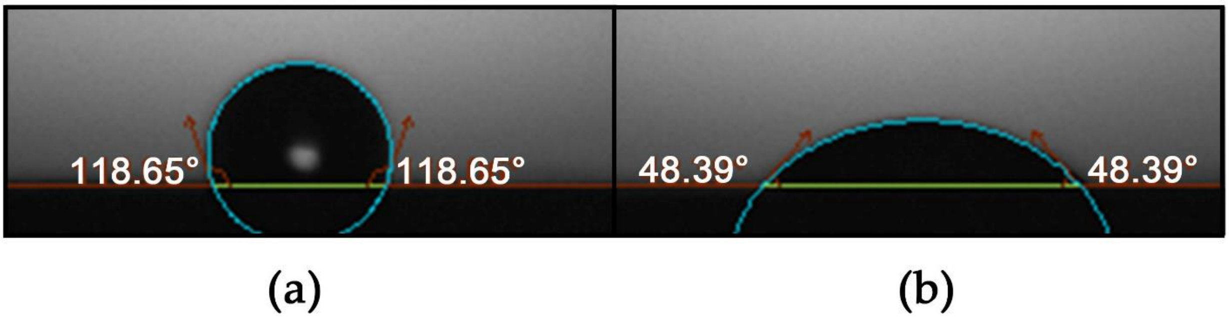

Figure S1. Contact angle analysis of (a) bare carbon electrode and (b) diazonium grafted surface.

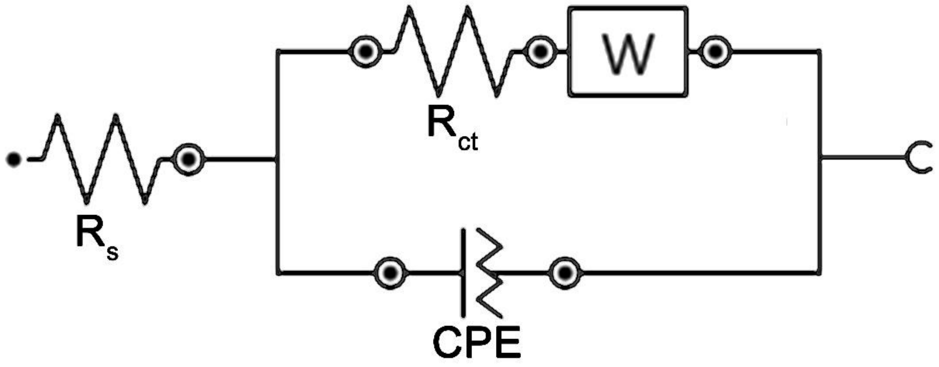

Figure S2. Suggested equivalent circuit model of the Nyquist plot from EIS measurements. R_s: solution resistance; R_{ct}: charge-transfer resistance; W: Warburg impedance; CPE: constant phase element.

Table S1. Fitting parameters for Nyquist plot for stepwise modification of the developed sensor.

Surface	EIS parameters	Fitted value	Fitted error (%)
Bare SPCE	Solution resistance, R_s	156.36 Ω	0.657
	Charge transfer resistance, R_{ct}	1568.10 Ω	0.58
	Constant phase element, CPE	0.92 µMho	4.035
	Warburg impedance, W	0.0013 Mho	1.317
SPCE-ABA	Solution resistance, R_s	164.72 Ω	0.36
	Charge transfer resistance, R_{ct}	49371 Ω	0.395
	Constant phase element, CPE	1.19 µMho	0.844
	Warburg impedance, W	0.0010 Mho	11.418

SPCE-ABA-EDC/NHS	Solution resistance, R_s	244 Ω	1.506
	Charge transfer resistance, R_{ct}	1562.20 Ω	1.122
	Constant phase element, CPE	23.56 μMho	3.391
	Warburg impedance, W	0.0013 Mho	1.448
SPCE-ABA-EDC/NHS-Aptamer	Solution resistance, R_s	154.68 Ω	0.427
	Charge transfer resistance, R_{ct}	10010 Ω	0.486
	Constant phase element, CPE	1.75 μMho	0.225
	Warburg impedance, W	0.0015 Mho	5.507
SPCE-ABA-EDC/NHS-Aptamer-Ethanolamine	Solution resistance, R_s	158.97 Ω	0.512
	Charge transfer resistance, R_{ct}	2224.50 Ω	0.548
	Constant phase element, CPE	3.31 μMho	2.691
	Warburg impedance, W	0.0014 Mho	1.473
SPCE-ABA-EDC/NHS-Aptamer-Ethanolamine-BSA	Solution resistance, R_s	133.9 Ω	1.799
	Charge transfer resistance, R_{ct}	3076.1 Ω	2.342
	Constant phase element, CPE	19.44 μMho	6.565
	Warburg impedance, W	0.0014Mho	6.291
